# Parkinson's Disease: The Mitochondria-Iron Link

**DOI:** 10.1155/2016/7049108

**Published:** 2016-05-17

**Authors:** Yorka Muñoz, Carlos M. Carrasco, Joaquín D. Campos, Pabla Aguirre, Marco T. Núñez

**Affiliations:** Iron and Biology of Aging Laboratory, Department of Biology, Faculty of Sciences, Universidad de Chile, Santiago, Chile

## Abstract

Mitochondrial dysfunction, iron accumulation, and oxidative damage are conditions often found in damaged brain areas of Parkinson's disease. We propose that a causal link exists between these three events. Mitochondrial dysfunction results not only in increased reactive oxygen species production but also in decreased iron-sulfur cluster synthesis and unorthodox activation of Iron Regulatory Protein 1 (IRP1), a key regulator of cell iron homeostasis. In turn, IRP1 activation results in iron accumulation and hydroxyl radical-mediated damage. These three occurrences—mitochondrial dysfunction, iron accumulation, and oxidative damage—generate a positive feedback loop of increased iron accumulation and oxidative stress. Here, we review the evidence that points to a link between mitochondrial dysfunction and iron accumulation as early events in the development of sporadic and genetic cases of Parkinson's disease. Finally, an attempt is done to contextualize the possible relationship between mitochondria dysfunction and iron dyshomeostasis. Based on published evidence, we propose that iron chelation—by decreasing iron-associated oxidative damage and by inducing cell survival and cell-rescue pathways—is a viable therapy for retarding this cycle.

## 1. Introduction

Parkinson's disease (PD) is the most frequent neurodegenerative movement disorder worldwide. Despite substantial amount of research, its founding causes remain elusive. Hence, while the initial causes of PD are not clearly determined, factors like aging, mitochondrial dysfunction, oxidative stress, and inflammation, are thought to have a pathogenic role in the disease [1–8]. PD is characterized by degeneration of dopaminergic neurons of the* substantia nigra pars compacta *(SNpc) and the presence of proteinaceous cytoplasmic inclusions, called Lewy bodies [9, 10]. Loss of dopaminergic neurons in the SNpc produces a decrease in dopamine levels in the* corpus striatum* generating a deregulation of basal ganglia circuitries that leads to the appearance of motor symptoms including resting tremor, rigidity, bradykinesia, and postural instability. In addition, nonmotor symptoms such as depression, cognitive deficits, gastrointestinal problems, sleep disturbances, and smell loss have been identified. Sporadic cases represent more than 90% of total PD patients, but there are several inherited forms caused by mutations in single genes. Although sporadic and familial PD cases have similar outcomes, inherited forms of the disease usually begin at earlier ages and are associated with atypical clinical features [11].

Mitochondrial dysfunction is a plausible cause of PD neurodegeneration. Endogenous and exogenous mitochondrial toxins like nitric oxide, 4-hydroxynonenal, aminochrome, paraquat, rotenone, and others have been linked to sporadic forms of the disease [7, 12–16], and mitochondrial defects have been described in SNpc mitochondria of PD patients [17, 18]. Additionally, as discussed below, several PD-associated proteins, including *α*-synuclein (*α*-syn), Parkin, PTEN-induced putative kinase 1 (PINK1), protein deglycase DJ-1, leucine-rich repeat kinase 2 (LRRK2), and P-type ATPase A2 (ATP13A2), point to a role for mitochondria in the development of the disease.

In another aspect of PD neurodegeneration, a large body of literature strongly indicates that excess redox-active iron is involved in the pathogenesis of PD [19–34]. Iron, in its ferrous (Fe^2+^) and ferric (Fe^3+^) states, is present in Lewy bodies as well as in many other amyloid structures [35–37]. Iron content in the SNpc is higher than in other areas of the brain [38] and is even higher in PD patients [39]. Here, we review the evidence that points to mitochondrial dysfunction and the subsequent iron accumulation as early events in the development of PD.

## 2. Cell Iron

Iron has been described as an important cofactor in many proteins involved in crucial biological processes, including cellular respiration, nitrogen fixation, photosynthesis, DNA synthesis and repair, oxygen transport, metabolism of xenobiotics, and neurotransmitter synthesis [40–49]. In most proteins iron is present in iron-sulfur clusters (ISCs), either as [2Fe-2S], [4Fe-4S], or [3Fe-4S] clusters [50, 51]. The main feature of iron as prosthetic group resides in its high redox flexibility. Thus, iron has the capacity to exchange one electron, either by oxidation (Fe^2+^ → Fe^3+^) or by reduction (Fe^3+^ → Fe^2+^). This flexibility is very important in biological processes such as cellular respiration, where the transport of electrons depends on 12 ISCs present in complex I to complex III and on 5 heme-containing proteins transporting electrons through complexes III and I [52].

Increases in redox-active iron directly associate with increased reactive oxygen species (ROS) generation and with changes in the intracellular reduction potential due to glutathione oxidation [53, 54]. Within the cell, most iron is associated with proteins, as either iron oxy-hydroxy crystals in ferritin or forming part of ISCs and heme prostetic groups. Around 1% of cell iron is in a redox-active form called the labile iron pool or labile cell iron [55–58]. The predominant component of this pool is Fe^2+^-glutathione, but iron is also bound weakly to phosphate, citrate, carboxylates, carbohydrates, nucleotides, polypeptides, and other molecules [59, 60]. Through the Fenton reaction, reactive iron catalyzes the production of hydroxyl radical (^∙^OH) in the presence of H_2_O_2_, in a self-renewed cycle caused by the presence of oxygen as an electron acceptor and intracellular reductants such as glutathione (GSH) and ascorbate as electron donors [28]. These characteristics of the intracellular environment demand a tight regulation of the reactive iron pool to decrease hydroxyl radical production.

Redox-active iron mediates GSH consumption [54]. After exposure to increasing concentrations of iron, SH-SY5Y dopaminergic cells undergo sustained iron accumulation and produce a biphasic change in intracellular GSH levels, increasing GSH levels at low iron concentrations and decreasing them thereafter. Indeed, cell exposure to high iron concentrations markedly decreases the GSH/GSSG molar ratio and the GSH half-cell reduction potential, with the associated loss in cell viability [54].

Iron levels in the SNpc increase significantly with age, and PD patients present an even greater increase that correlates with clinical PD status [64–69]. Experimental evidence shows that iron is crucial to the degeneration of SNpc dopaminergic neurons in the model of PD caused by 1-methyl-4-phenyl-1,2,3,6-tetrahydropyridine (MPTP). Mice fed for 6 weeks with a low iron diet before the administration of MPTP present neuronal protection, normal striatal dopamine levels, and no changes in motor behavior when compared with control animals fed a normal iron content diet [70]. Furthermore, increased iron levels in the brain aggravate dopaminergic cell death and motor impairment after MPTP treatment and this condition is attenuated by treatment with the iron chelator desferrioxamine (DFO) [71].

Clinical studies have not provided an evident correlation between dietary iron intake and risk of Parkinson's disease in humans [72–75]. Nevertheless, some reports point to a higher incidence of PD in hereditary hemochromatosis patients [76–79] although other reports found no correlation between these two diseases [80–82]. It is possible that under normal conditions the iron homeostasis system protects the brain from iron accumulation due to dietary variations. This homeostasis is most likely lost in iron-overload disease states yet.

Overall, these antecedents suggest that increased redox-active iron in the SNpc is part of the neurodegenerative process in PD, possibly due to increased oxidative stress and oxidative damage.

## 3. Iron Homeostasis in Mitochondria

Mitochondria consume about 90% of cellular oxygen and transform 1–5% of this oxygen into superoxide anion (O_2_^∙−^), due to the leaking of electrons that takes place in their passage through complexes III and I [83–86]. During aging, the activity of these complexes decreases, leading to higher oxidant production of O_2_^∙−^ and H_2_O_2_ [86, 87]. The superoxide anion generated in this process dismutates into hydrogen peroxide, either spontaneously or following catalysis by superoxide dismutase (SOD) [88, 89]. Proteins containing ISCs in mitochondria are significantly vulnerable to oxidative stress, participating in redox sensing and signaling reactions [90, 91].

**Figure 1:**
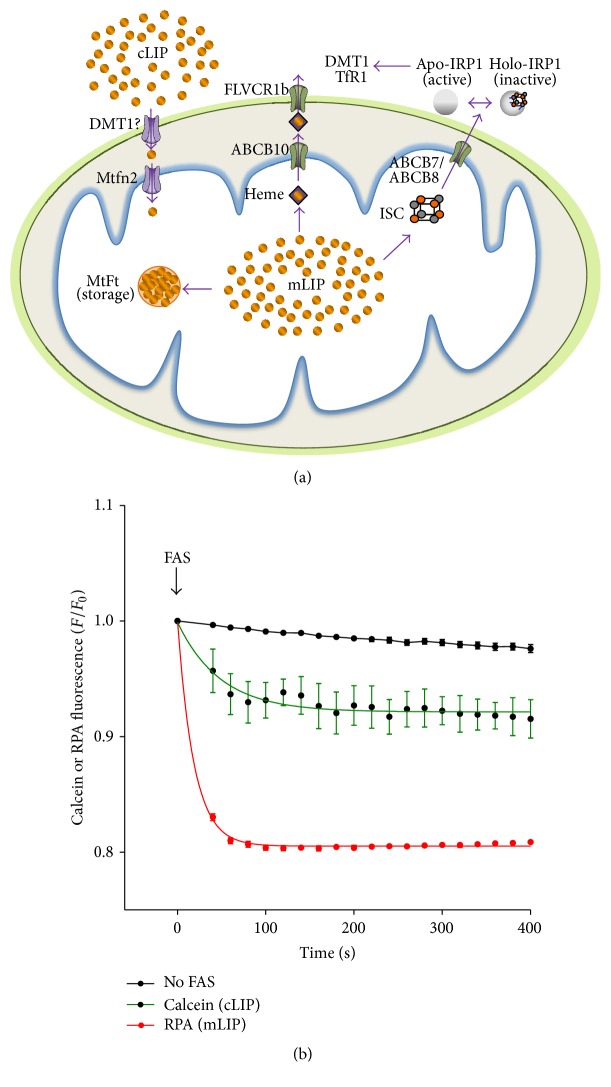
(a) Mitochondrial iron traffic. Iron enters mitochondria from the cLIP in a process mediated by the inner mitochondrial iron transporter Mtfn2 and probably by DMT1 located in the outer membrane. Upon entering, iron incorporates into the mLIP from where it distributes for heme and ISC synthesis or for storage in mFt. Heme leaves the mitochondrion through ABCB10 and the mitochondrial heme exporter FLVCR1b, located in the inner and outer mitochondrial membranes, respectively. ISCs are transported out of the mitochondrion by the ABCB7 transporter and probably by the ABCB8 transporter as well. In the cytoplasm, ISCs bind to the corresponding apoproteins. IRP1 binds a 4Fe-4S cluster; the holoprotein is inactive to induce the transcriptional regulation of cell iron-import proteins like DMT1 and TfR1. In contrast, apo-IRP1, normally abundant under low cell iron conditions, upregulates the expression of iron-import proteins like DMT1 and TfR1. ABC: ATP-binding cassette transporter; cLIP: cytoplasmic labile iron pool; DMT1: divalent metal transporter 1; FLVCR1b: feline leukemia virus subgroup C receptor 1B transporter; ISC: iron-sulfur cluster; mFt: mitochondrial ferritin; mLIP: mitochondrial iron pool; Mtfn2: mitoferrin-2; TfR1: transferrin receptor 1. (b) Kinetic determination of iron entrance into the cLIP and mLIP. SH-SY5Y cells preloaded with the mitochondrial iron sensor rhodamine B-[(1,10-phenanthroline-5-yl)aminocarbonyl]benzyl ester (RPA) and the cytoplasmic iron sensor calcein were challenged with 40 *μ*M ferrous ammonium sulfate (Fe) and changes in RPA and calcein fluorescence were followed in a multiplate fluorescence reader [61, 62]. Iron binding quenches RPA and calcein fluorescence; thus, a decrease in RPA or calcein fluorescence is directly proportional to iron entrance into the mLIP or cLIP, respectively. Note that the initial rate of iron entrance into the mLIP (*K* = 0.0536 ± 0.0021Δ(*F*/*F*_0_)/sec) is larger than the rate of iron entrance into the cytoplasmic LIP (*K* = 0.0206 ± 0.0070Δ(*F*/*F*_0_)/sec). Values represent mean ± SD of quadruplicates; *P* = 0.004.

The mitochondrion has an active exchange of iron with the cytoplasm, as required for the mitochondrial synthesis of heme and ISCs (Figure 1(a)) [92–94]. Kinetic experiments show that extracellular iron is readily incorporated into mitochondria. Indeed, iron incorporation into mitochondria apparently has a kinetic preference over incorporation into the cytoplasm (Figure 1(b)) (also see [94, 95]). Possible mechanisms for this preferential delivery include siderophore-mediated iron transport from the plasma membrane to the mitochondrion [96, 97], the entrance of iron into the cell by fluid-phase endocytosis with subsequent delivery to mitochondria without passing through the cytoplasmic labile iron pool (cLIP) [98], and iron delivery to mitochondrion by direct interaction with transferrin-containing endosomes [99].

Mitoferrin-2, a protein located in the inner mitochondrial membrane, represents the main pathway of mitochondrial iron uptake, whereas the ABCB7 and ABCB8 transporters are involved in ISC export [100–103] (Figure 1). Inward transport of iron by mitoferrin-2 apparently is regulated. Studies with the mitoferrin Mrs3p and Mrs4p yeast homologs revealed that inner mitochondrial membrane vesicles show rapid uptake of Fe^2+^ in response to iron starvation [104]. There is no reported evidence as to how cell or mitochondrial iron levels could regulate mitoferrin-2 levels. Additionally, mitoferrin dysregulation under pathological conditions promotes mitochondrial iron accumulation [100, 104].

A recent report described a role for mitoferrin-2 in the development of Friedreich's ataxia, by showing that mitoferrin-2 downregulation improved many of the conditions of frataxin deficiency whereas its overexpression exacerbated them [105]. Similarly, loss-of-function mutations in* ABCB7* produce a sideroblastic anemia condition called X-chromosome-linked sideroblastic anemia, in which patients show iron accumulation in mitochondria [101, 102].

**Figure 2:**
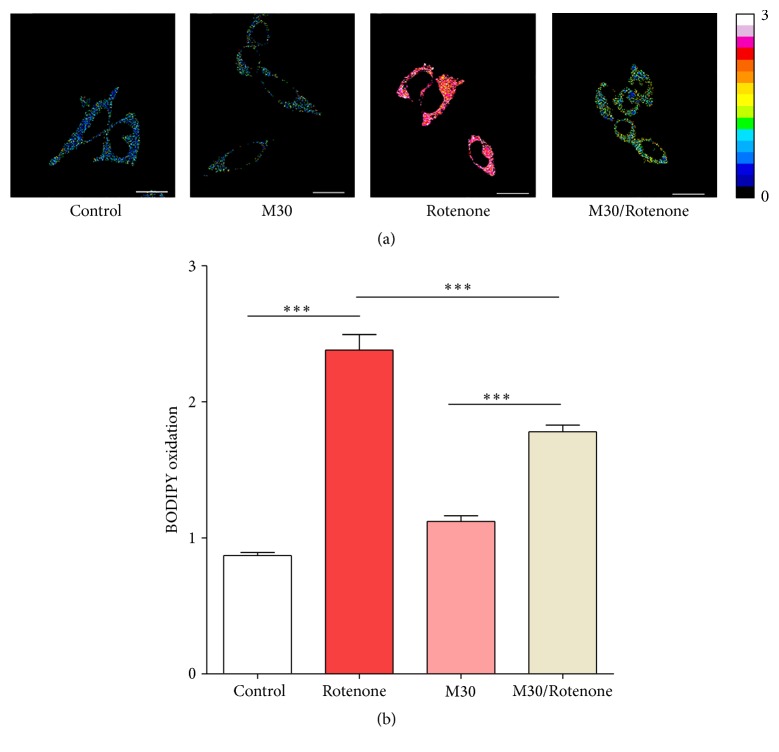
The iron Chelator M30 protect SH-SY5Y cells from rotenone-induce lipid peroxidation. (a) Mitochondrial lipid peroxidation was evaluated by green/red fluorescence changes of C11-BODIPY^581/591^ (ThermoFisher Scientific-Molecular Probes) as described [63]. Oxidation of C11-BODIPY^581/591^ results in a shift of the fluorescence emission peak from 590 nm (red, nonoxidized) to 510 nm (green, oxidized). SH-SY5Y cells were preincubated or not for 24 hours with 500 nM of M30 in DMEM-10% FCS medium and then loaded for 15 minutes at 37°C with 1 *μ*M C11-BODIPY^581/591^. Confocal images were obtained 15 minutes both before (Control, M30) and after (Rotenone, M30/Rotenone) applying 80 *μ*M rotenone to the cells. Representative images are shown, where the ratio of green over (green + red) fluorescence was converted into a pseudothermal scale using the ImageJ program. (b) Changes in C11-BODIPY^581/591^ oxidation quantified by the thermal scale. Values represent the mean ± SD of 40–52 individual cell measures per experimental condition. Significance between mean differences was determined by one-way ANOVA and Tukey* post hoc* test. ^*∗∗∗*^ *P* < 0.001.

A fraction of the intramitochondrial iron is redox-active. Petrat et al. demonstrated presence of a chelatable iron pool, which renders mitochondria sensitive to iron-mediated oxidative damage [106]. Evidence from our laboratory shows that complex I inhibition generates mitochondrial lipid peroxidation as determined by C11-BODIPY^581/591^ oxidation [63], which is probably caused by redox-active iron since it is inhibited by coincubation with the iron chelator M30 (Figure 2).

## 4. Mitochondrial Dysfunction in PD

Mitochondrial dysfunction and oxidative stress have long been implied as pathophysiological mechanisms underlying PD [17, 107]. Mitochondria not only have a key role in electron transport and oxidative phosphorylation but also are the main cellular source of ROS and they are involved in calcium homeostasis and in the regulation and initiation of cell death pathways [1]. Mitochondria isolated from human brain tissues and peripheral cells of sporadic PD patients exhibit reduced mitochondrial complex I activity [108] and postmortem SNpc tissues from idiopathic PD patients display decreased number of complex I subunits [107, 109, 110]. Mitochondrial complex I activity is reduced in the SNpc [111] and the frontal cortex [112] in patients with PD. However, total protein and mitochondrial mass from SNpc of patients with PD are similar to controls [111]. The main consequences of mitochondrial complex I inhibition in humans and experimental models are decreased ATP levels [113, 114], decreased glutathione levels, and increased oxidative damage [115–118]. Other reported effects are reduction in the concentrations of DA accompanied with decreased density of DA receptors and diminished activity of TH (reviewed in [119]), increased total SNpc iron content [120], increased redox-active iron [121, 122], decreased Fe-S cluster synthesis [61, 123], and calcium dysregulation [124–126]. Any one of these events may result in cell death once the homeostatic mechanisms are surpassed.

The first evidence of mitochondrial dysfunction as a causal source of PD was obtained in the 1980s when four students developed marked Parkinsonism after intravenous injection of an illicit drug contaminated with MPTP. Because of the striking Parkinson-like features and additional pathological data, it was proposed that MPTP selectively damaged dopaminergic neurons in the SNpc causing the Parkinson syndromes [127]. Later studies showed that MPTP causes an irreversible destruction of the dopaminergic nigrostriatal pathway that results in symptoms of Parkinsonism in primates and mice [128–130].

In animal models of PD, inhibition of complex I by MPTP or 6-hydroxydopamine (6-OHDA) results in iron accumulation in the SNpc [131, 132]. Importantly, iron chelators effectively abrogate this neurodegenerative process (see below). Thus, with all probability redox-active iron mediates the degenerative process of SNpc neurons induced by inhibition of complex I.

## 5. IRP1: The Link between Mitochondrial Dysfunction and Iron Dyshomeostasis

Iron Regulatory Proteins 1 and 2 (IRP1 and IRP2) are largely responsible for maintaining cytoplasmic iron levels through the translational regulation of iron homeostasis proteins. IRPs bind to RNA stem loops called iron responsive elements (IREs), which are found in untranslated regions of target mRNAs that encode proteins involved in iron metabolism. Binding of IRPs to IREs in the 5′-untranslated region inhibits the translation of mRNA, as is the case for the iron-storage protein ferritin. Binding of IRPs to IREs present in the 3′-untranslated region increases the stability of mRNAs, thus increasing the translation of DMT1 and the transferrin receptor [133, 134].

Importantly, IRP1 activity depends on the protein having or not a 4Fe-4S cluster. Binding of the 4Fe-4S cluster to IRP1 renders the protein inactive to bind to mRNA [135]. Low cell iron induces the dissociation of this 4Fe-4S cluster activating IRP1 and inducing the expression of iron uptake proteins like the transferrin receptor 1 (TfR1) and dimetal iron transporter 1 (DMT1) [136]. Furthermore, IRP1 is sensitive to several oxidative stress stimulus: hydrogen peroxide, nitric oxide, and peroxynitrite all activate IRP1 by induction of ISC disassembly [137, 138], while superoxide inhibits aconitase activity [139].

IRP1 is deregulated in PD tissue, since postmortem brain tissue from PD patients displays increased IRP1 activity when compared to tissue from control individuals. Increased IRP1 activity was found also in the ipsilateral ventral mesencephalon of 6-OHDA-treated rats [140]. Studies performed in our laboratory showed that in SH-SY5Y cells inhibition of complex I by rotenone results in decreased Fe-S cluster synthesis and increased IRP1 mRNA binding activity, accompanied by increased cLIP [61]. Therefore, inhibition of complex I and the subsequent activation of IRP1 lead to increased DMT1 and TfR1 expression, increased iron uptake, and increased ROS generation.

## 6. Environmental Toxicants, Mitochondrial Dysfunction, and Iron Dyshomeostasis

A considerable body of evidence epidemiologically links exposure to environmental toxicants like paraquat and rotenone to the generation of PD in rural workers [141–144]. The herbicide paraquat is a free radical generator that inhibits mitochondrial electron-transport activity [145–147] and causes dopaminergic neuron loss, *α*-synuclein aggregation, and motor deficits in rodents, with a dramatic increase in free radical formation [148–150]. Moreover, systemic application of paraquat reduces motor activity and induces dose-dependent loss of striatal tyrosine hydroxylase positive (TH+) fibers and SNpc neurons in mice [151–154]. Paraquat has been proposed to cause Parkinsonism in humans. However, the clinical and epidemiological evidence in this regard is still inconclusive [1, 144, 155, 156]. In fact, paraquat remains one of the most widely used herbicides in developing countries [157, 158].

Although its association with PD is not firmly established, emerging evidence links paraquat exposure to brain iron accumulation. Patients from acute paraquat poisoning displayed excessive brain iron deposition [159]. Similarly, incubation of rat primary mesencephalic cultures with paraquat resulted in increased production of H_2_O_2_ and Fe^2+^ at times preceding cell death [160]. Mechanistic studies identified m-aconitase from astrocytes as the main mediator in ROS production, although neurons were identified as the primary dying cell type, and death was attenuated by addition of catalase and/or a cell permeable iron chelator [160]. We propose that these results are consistent with a mechanism whereby paraquat affects mitochondrial activity resulting in increased ROS production and increased iron content, a combination that induces neuronal death by hydroxyl radical-mediated damage.

Rotenone is a classic complex I inhibitor [161, 162]. Both rotenone and MPP+ inhibit complex I NADH dehydrogenase, shutting off mitochondrial respiration and causing selective injury of SNpc neurons [128, 163–166]. Rotenone and MPP+ also produce superoxide anion in submitochondrial particles [167–169]. Chronic rotenone administration to mice reproduces Parkinson-like syndromes that include death of SNpc neurons, complex I inhibition, and Lewy bodies-like fibrillar cytoplasmic inclusions containing ubiquitin and *α*-synuclein [141, 170].

Treatment with rotenone induces iron accumulation in animal and cell models [61, 171]. Rats treated with rotenone evidence iron accumulation in the SNpc, the striatum, the globus pallidus, and other brain areas and treatment with iron chelating agents significantly reduces iron deposition and the loss of dopaminergic neurons in these areas [171]. Similarly, treatment of SH-SY5Y dopaminergic neuroblastoma cells whit rotenone results in mitochondrial iron accumulation and oxidative damage [172]. The mitochondria-tagged iron chelator Q1 abolishes both effects [94]. Overall, these data are consistent with the notion that inhibition of complex I results in the dysregulation of iron homeostasis in dopaminergic cells.

In summary, although the epidemiological evidence that links paraquat or rotenone exposure with PD still needs consolidation, increasing evidence shows that inhibition of mitochondrial activity by these compounds results in iron accumulation. The mechanisms causing this accumulation are unknown. Considering the previous* in vitro* evidences discussed above, iron accumulation may be mediated by activation of IRP1 due to decreased ISC synthesis.

## 7. PD Genes Associated with Mitochondrial Dysfunction and Iron Accumulation

As detailed below, a wealth of reports indicate that the product of a number of PD-associated genes, including *α*-syn, Parkin, PINK1, DJ-1, LRRK2, and ATP13A2, disrupts mitochondrial function. Moreover, this disruption is generally associated with increased iron load. Here we will review the evidence that links mitochondrial dysfunction and iron accumulation in familial cases of PD.

### 7.1. *α*-Syn

The function of wild type *α*-syn is still an open issue [173, 174]. There is consensus, however, that misfolding and aggregation of *α*-syn underlie its toxicity in both PD and Lewy body-associated dementia [173]. Accumulation of cytosolic *α*-syn can render toxic endogenous dopamine [175] and acts as a seed promoting the formation of cytosolic inclusions [176]. If degradation pathways do not clear these aggregates promptly, neurodegeneration can ensue.

There is a reciprocal relationship between *α*-syn activity and mitochondrial function; thus, *α*-syn overexpression in dopaminergic cell lines results in mitochondrial alterations accompanied by increased levels of ROS [177–180]. The N-terminal sequence of *α*-syn contains a cryptic mitochondrial targeting signal, and *α*-syn has been localized into mitochondria after acidification of the cytosol or *α*-syn overexpression [181, 182]. Mitochondrial *α*-syn decreases the activity of complex I, increases ROS production [183], causes cytochrome c release, increases mitochondrial calcium and nitric oxide levels, and induces oxidative modification of mitochondrial components [184]. Moreover, mice that overexpress *α*-syn A53T exhibit dysmorphic mitochondria with evidence of DNA damage [185], while administration of MPTP to mice that overexpress *α*-syn leads to swollen and morphologically abnormal mitochondria [186]. An open issue is whether *α*-syn aggregation promotes mitochondrial dysfunction or vice versa. Probably both phenomena are interrelated: *α*-syn induces mitochondrial dysfunction and mitochondrial dysfunction induces *α*-syn aggregation [187].

Recent evidence suggests that *α*-syn aggregation induces iron accumulation. In PD patient brains, neurons containing *α*-syn deposits also display increased iron concentrations and upregulated levels of Nedd4 Family Interacting Protein 1 (Ndfip1), an adaptor for the neuronal precursor cell-expressed developmentally downregulated 4 (Nedd4) family of E3 ligases [188]. Similarly, rat midbrain neurons and PC12 cells overexpressing human *α*-syn accumulate increased levels of iron and show iron redistribution from the cytoplasm to the perinuclear region within *α*-synuclein-rich inclusions [189].

Interactions between iron and *α*-syn most probably contribute to the process of neurodegeneration [190]. Further work indicated that divalent metals, including Fe^2+^, Mn^2+^, Co^2+^, and Ni^2+^, bind to the C-terminal of *α*-syn, and the N-terminus residues 119–124 were recognized as the main binding site of divalent metal ions [191]. Incubation of wild type and mutant *α*-syn with Fe^3+^ resulted in the formation of short thick fibrils [192]. In BE(2)-M17 cells overexpressing wild type or mutant *α*-syn (A30P and A53T), treatment with Fe^2+^, dopamine, and hydrogen peroxide generated *α*-syn-positive inclusions, which also contained ubiquitin [193]. Similarly, Fe^2+^-treated BE(2)-M17 cells were more susceptible to Fe^2+^-induced DNA damage when overexpressing mutant *α*-syn [194]. In contrast, Mg^2+^ inhibits both spontaneous and Fe^2+^-induced aggregation of wild type but not A53T *α*-syn [195], and dopamine suppresses the Fe^3+^-induced fibrillation of *α*-syn [196].

Interestingly, *α*-syn aggregation in turn produces oxidative stress, in a process mediated by metal ions like Fe and Mn, thus generating a vicious cycle between oxidative stress and *α*-syn aggregation [197–201]. Moreover, pesticides such as rotenone, paraquat and dieldrin, and metal ions (iron, manganese, copper, lead, mercury, zinc, and aluminum) induce a conformational change in *α*-syn and directly accelerate the rate of formation of *α*-syn fibrils* in vitro* [202–204]. In addition, the simultaneous presence of metal ions and pesticides leads to synergistic effects on the rate of fibrillation [205].

In summary, there seems to be a cyclic association between *α*-syn and iron in which *α*-syn induces iron accumulation and iron induces *α*-syn aggregation. This cycle is aggravated by *α*-syn-induced mitochondrial dysfunction. These associations may originate a sequence of events in which *α*-syn aggregation induces mitochondrial dysfunction, which in turn results in iron accumulation and further *α*-syn aggregation and hydroxyl radical-mediated damage.

### 7.2. Parkin

Various mutations in Parkin, an E3 ubiquitin ligase of the ubiquitin-proteasome system, lead to an autosomal recessive PD form, which also is seen in some young-onset sporadic PD cases [206, 207]. Abundant evidence links Parkin to mitochondrial function. Cultured fibroblasts from patients carrying Parkin mutations present longer and more branched mitochondria than controls [208] and leukocyte mitochondrial complex I and IV activities are reduced in PD patients who are homozygous for Parkin mutations [209]. Parkin-deficient mice have decreased levels of mitochondrial complexes I and IV in the striatum, together with increased protein and lipid peroxidation [210]. In addition, Parkin-null* D. melanogaster *mutants develop muscle degeneration with mitochondrial pathology and display decreased resistance to oxidative stress [211, 212]. Moreover, overexpression of Parkin attenuates the dopaminergic neurodegeneration induced by MPTP through protection of mitochondria and reduction of *α*-syn in the nigrostriatal pathway [213]. After chronic MPTP administration, Parkin overexpression prevents motor deficits and dopaminergic cell loss in mice [214].

Published observations linking Parkin mutations and iron accumulation are scarce. In an initial study, PD patients carrying Parkin mutations as well as mutation carriers without clinical manifestations of the disease showed increased echogenicity of the SNpc, which in asymptomatic Parkin mutation carriers was associated with abnormal nigrostriatal F-dopa positron emission tomography [215, 216]. Recently, a R2^*∗*^ relaxometry study in the SNpc of genetic and idiopathic PD patients reported that R2^*∗*^ values, indicative of iron deposition, were increased in idiopathic PD patients and in patients carrying Parkin and LRRK2 mutations when compared to control subjects [217].

Overall, the bulk of the evidence points to a relationship between Parkin and mitochondria structural functionality. Further investigations are needed to assert if PD Parkin mutations also result in iron dyshomeostasis.

### 7.3. PINK1

Mutations in PINK1, a serine-threonine protein kinase localized to the mitochondrial membrane via an N-terminal mitochondrial targeting sequence [218], lead to a rare autosomal form of PD. It is generally accepted that PINK1 has a physiological role in mitochondria maintenance, suppressing mitochondrial oxidative stress, fission, and autophagy [219]. PINK1 KO mice exhibit age-dependent moderate reduction in striatal dopamine levels, accompanied by low locomotor activity [220–222]. These mice show no loss of dopaminergic neurons in the SNpc region but display decreased striatal innervations [223, 224], together with decreased mitochondrial respiration and mitochondrial aconitase activity in the striatum [220].

Fibroblasts from patients homozygous for the G309D-PINK1 mutation have reduced complex I activity and evidence oxidative damage compared with cells from control individuals [225]. In flies, PINK1 deficiency results in loss of dopaminergic cells, enhanced susceptibility to oxidative stress, reduced mitochondrial mass with disorganized morphology, and decreased ATP levels [226]. Parkin and PINK1 work in a common pathway, with Parkin acting downstream of PINK1 [226–228]. Under conditions of severe mitochondrial damage, PINK1 and Parkin act to induce mitophagy and mitochondrial membrane depolarization [229]. PINK1 also regulates mitochondrial dynamics through interaction with the fission/fusion machinery [230]. Further genetic studies in* Drosophila* revealed that the PINK1/Parkin pathway regulates mitochondrial morphology by tipping the balance of mitochondrial fission/fusion dynamics toward fission in dopaminergic and hippocampal neurons [230, 231] and muscle cells [232–234].

In SNpc dopaminergic neurons, PINK1 is required to maintain normal mitochondrial morphology and membrane potential, exerting this neuroprotective effects by inhibiting ROS formation [235]. In human dopaminergic neurons, PINK1 deficiency produces mitochondrial dysfunction and marked oxidative stress. These defects result in reduced long-term cell viability, with neurons dying via cytochrome c-mediated apoptosis [236]. Additionally, PINK1 knockdown SH-SY5Y cells show decreased resistance against thapsigargin-induced apoptosis, while PINK1 overexpression restores it [237].

Evidence linking PINK1 and iron is scarce. Patients carrying a PINK1 mutation display a significantly larger area of SNpc echogenicity assessed with transcranial ultrasound relative to healthy controls [238]. In a* Drosophila* model, PINK1 mutants present increased superoxide levels, which induce 4Fe-4S cluster inactivation and increased iron levels in the mitochondrion [239]. As discussed above, decreased ISC synthesis can lead to iron accumulation through IRP1 activation [61].

Overall, published data indicates that under conditions of PINK1 deficiency mitochondrial quality control mechanisms are compromised, resulting in increased ROS production and apoptotic cell death. Up to date, evidence of a relationship between PINK1 loss of function and iron dyshomeostasis is discrete but enticing. The observation of decreased mitochondrial aconitase activity, indicative of a possible decrease in ISC synthesis, and the observed link between PINK1 mutations and superoxide-mediated iron accumulation in mitochondria are powerful incentives to study possible changes in iron homeostasis under PINK1 deficiency and to assess how these changes impact on cell death.

### 7.4. DJ-1

DJ-1 is a multitask protein that participates in the protection of cells from oxidative stress-related death [240–243]. DJ-1* null *mice show decreased locomotor activity, a reduction in the release of evoked dopamine in striatum but no loss of SNpc dopaminergic neurons [223, 224]. A relationship between DJ-1 and mitochondrial function has long been suspected [244]; however, DJ-1-null mice show no apparent mitochondrial defects [223, 224]. In contrast, ROS production, mitochondrial structural damages, and complex I deficit are significantly higher in DJ-1-null cultured dopaminergic neurons [245].

To date, the evidence linking DJ-1 and iron is scanty. PD patients carrying DJ-1 mutations have an area in the SNpc of significantly larger echogenicity than in healthy controls [238]. As SNpc hyperechogenicity is related to increased iron content, these findings suggest that DJ-1 mutations may result in iron accumulation.

### 7.5. LRRK2

LRRK2 is a cytosolic serine-threonine-protein kinase, with a fraction of about 10% associated with the outer mitochondria membrane. Overall, LRRK2 mice models display mild or no functional disruption of nigrostriatal dopaminergic neurons of the SNpc [246]. Recently, a new LRRK2 knock-in mice evidenced profound mitochondrial abnormalities in the striatum of older homozygous mice, which are consistent with mitochondrial fission arrest described previously [247]. In skin biopsies from human LRRK2 G2019S carriers, however, mitochondrial function and morphology are perturbed, as demonstrated by reduced mitochondrial membrane potential, reduced intracellular ATP levels, mitochondrial elongation, and increased mitochondrial interconnectivity [248]. LRRK2 mutations reduce the activity of peroxiredoxin 3, an antioxidant enzyme located within mitochondria. This effect appears to be phosphorylation-dependent [249, 250].

To date, just a few studies have shown a relationship between LRRK2 dysfunction and iron accumulation. In a recent study determining R2^*∗*^ relaxometry rate, high nigral iron deposition in LRRK2 mutation carriers was demonstrated [217]. In a small cohort of patients, it was found that R2^*∗*^ values in the SNpc were increased in idiopathic PD patients and LRRK2 mutation-carrying patients as compared with controls, with LRRK2 mutation patient having larger R2^*∗*^ values than idiopathic PD patients [217]. Similarly, studies using transcranial sonography showed that LRRK2-associated PD patients had increased iron levels in the SNpc [238, 251]. These evidences support the notion that PD resulting from a variation in the LRRK2 allele has an iron accumulation component that affects neurodegeneration via increased oxidative damage. Further analysis will be required to evaluate this hypothesis.

### 7.6. ATP13A2

ATP13A2 is a lysosomal P-type 5 ATPase. Mutations in its gene are associated with a juvenile-onset, levodopa-responsive PD type named familial Kufor-Rakeb syndrome [252, 253]. ATP13A2* null* mice display late-onset sensorimotor deficits and deposition of *α*-syn aggregates without changes in the number of dopaminergic neurons in the SNpc or in striatal dopamine levels [254]. Arguably, ATP13A2 may help prevent neurodegeneration both by inhibiting *α*-syn aggregation and by supporting normal lysosomal and mitochondrial function [253].

A relationship between ATP13A2 and mitochondrial function is emerging. Reduced activity of ATP13A2 mutants may lead to mitochondrial defects [255] and higher ROS levels [256]. Fibroblasts from Kufor-Rakeb syndrome patients show lower mitochondrial membrane potential and lower ATP synthesis rates than fibroblast from controls [257]. In addition, overexpression of ATP13A2 inhibits cadmium-induced mitochondrial fragmentation, while silencing ATP13A2 expression induces mitochondrial fragmentation [258]. It remains to be elucidated if ATP13A2-associated mitochondrial dysfunction is due to a primary effect of on mitochondria integrity or is secondary to other event(s), like increased *α*-syn aggregation.

Two recent studies report neurodegeneration with brain iron accumulation in one Pakistani [259] and one Chilean [257] Kufor-Rakeb syndrome patients. Both patients showed abnormal bilateral hypo intensity in the putamen and caudate nuclei on T2^∗^ diffuse MRI images. In the Pakistani patient case, the clinicians attributed the abnormal MRI hypo intensity to iron deposition [259]. In the Chilean patient, the clinicians attributed the hypo intensity to ferritin deposits though they did not perform tests to exclude the possibility of deposition of other metal ions [257]. However, another study reported opposite results in an adolescent Brazilian patient with homozygous ATP13A2 mutation [260]. It is possible that brain metal ion accumulation only occurs very late in the course of the disease or in cases in which ATP13A2 mutations lead to a total loss of protein function, such as the Pakistani patient described by Schneider et al. [259]. Additional studies in patients with pathogenic ATP13A2 mutations are needed to clarify this point.

In summary, the activities of several PD genes, namely, *α*-syn, Parkin, PINK1, DJ-1, LRRK2, and ATP13A2, are involved in the maintenance of mitochondrial function and integrity. Mutations in these genes that result in familial PD are accompanied by decreased mitochondrial activity and increased oxidative stress. Emerging evidence points to iron dyshomeostasis as a direct or indirect consequence of decreased mitochondrial activity. There is much to learn regarding the mechanisms linking particular mitochondria-associated PD proteins with iron dyshomeostasis.

The question arises on the reasons why dopaminergic neurons from SNpc are more sensitive to neurodegeneration than similar neurons in the midbrain. Neurons from SNpc have increased IRP1 activity [61, 123, 261] and increased DMT1 expression [262–264] coupled to decreased ferritin expression [265–267], which most probably results in increased redox-active iron and oxidative damage. Similarly, intrinsic L-type calcium channel pace-marker activity and the associated tendency to elevated calcium levels [268, 269] put a metabolic burden in these neurons. Both aspects, iron and calcium burden, are particular factors in SNpc neurons that could be augmented by mitochondrial dysfunction.

## 8. Iron, Mitochondrial Dynamics, and Mitophagy

Mitochondria are highly dynamic organelles that continuously fuse and divide through the processes of fusion and fission, respectively. Increases in the fission events generate fragmented mitochondria whereas fusion events produce elongated mitochondria. A balance between mitochondrial fusion and fission is important in cellular function [270] and an imbalance can promote neuronal dysfunction and cell death [269, 271]. In neurons, mitochondrial fission is crucial for axonal transport of the organelles into areas of high metabolic demand, whereas mitochondrial fusion supports substitution and regeneration of mitochondrial proteins, mitochondrial DNA repair, and functional recovery. Indeed, enhanced mitochondrial fragmentation was associated with induction of neuronal death triggered by oxidative stress [272].

Dynamin-related protein 1 (Drp1) is a key regulator of mitochondrial fission and it has been associated with neuronal cell death induced by glutamate toxicity or oxygen-glucose deprivation* in vitro* and after ischemic brain damage* in vivo* [273]. Many studies have demonstrated that posttranslational modification of Drp1 (phosphorylation, ubiquitination, S-nitrosylation, and others) affects Drp1 activity and contributes to altered mitochondria dynamics and neurodegeneration in cell culture systems [274–278]. Recently, it was shown that ferric ammonium citrate (FAC) decreased cell viability and promoted cell death of HT-22 cells [279]. The FAC-induced iron overload triggered mitochondrial fragmentation and Drp1(Ser637) dephosphorylation by calcineurin. Iron chelation and pharmacological inhibition of calcineurin prevented mitochondrial fragmentation and apoptotic death. These findings suggest that, under iron-induced toxicity, calcineurin-mediated dephosphorylation of Drp1(Ser637) mediates neuronal cell loss by modulating mitochondrial dynamics [279].

As mentioned above, several groups observed that a deficiency in Parkin and PINK1 leads to mitochondrial pathology [211, 234, 280, 281]. PINK1 overexpression suppressed the translocation of Drp1 from the cytosol to the mitochondria, maintaining mitochondrial function [282]. In Drp1-deficient cells the Parkin/PINK1 knockdown phenotype did not occur, indicating that mitochondrial alterations observed in Parkin- or PINK1-deficient cells are associated with an increase in mitochondrial fission [281]. Moreover, Drp1 seems to activate autophagy/mitophagy pathways for morphologic remodeling of mitochondria in PINK1-deficient neuroblastoma cells [283]. Currently, the inhibition of Drp1 has been proposed as a strategy of neuroprotection in many neurodegenerative diseases because the altered Drp1 activity promotes exacerbated mitochondrial fragmentation.

Iron induces calcium release from intracellular stores, increase that is mediated by the ryanodine receptor (RyR) calcium channel [284]. A recent study showed that in hippocampal neurons iron induced a RyR-dependent increase in mitochondria-associated Drp1 together with increased mitochondrial fragmentation [285]. These results suggest that iron accumulation contributes to mitochondrial fission and, presumably, to the impairment of neuronal function by a mechanism that involves RyR activation, calcium release, and Drp1 activation.

## 9. Iron Chelation as a Therapeutic Approach for the Treatment of PD

Iron chelators are molecules from different origins with the ability to coordinate iron ions. In general, three distinct groups are identified: siderophores isolated from lithotrophic bacteria, phytochemicals, and synthetic molecules. Historically, the clinical use of these chelators has been focused on the treatment of iron-overload syndromes such as hemochromatosis, *β*-thalassemia, myelodysplastic syndrome, and other blood transfusion-requiring diseases [286, 287]. As discussed above, however, during the last years a growing set of evidences has demonstrated that many neurodegenerative disorders, prominently PD, present an iron accumulation component in the affected brain areas [7, 288–292]. Desferrioxamine (DFO) in 6-OHDA intoxicated rats provided the first evidence of neuroprotection by iron chelation. Injection of DFO in one cerebral ventricle of rats previously intoxicated showed partial protection from depletion of DA in the striatum and improvement in behavioral tests with respect to the intoxicated rats without DFO administration [293]. Recently, intranasal administration of DFO to the *α*-syn rat model of PD decreased Fe^+3^ content and the number of *α*-syn inclusions but did not protect dopaminergic neurons from death [294]. Administration of DFO to endotoxin-shocked mice attenuates the inflammatory response by suppressing the activation of mitogen-activated protein kinase (MAPKs) and NF-*κ*B [295], suggesting an anti-inflammatory effect of DFO. This is a potentially important observation given that inflammation is associated with the dysregulation of iron homeostasis [296–298].

Given the positive effects of DFO and other chelators like clioquinol and deferiprone (DFP) in PD and other models of neurodegeneration [290, 299–301], a series of new 8-OH-quinoline-based chelators was developed, which include VK-28, HLA-20, M30, and VAR. VK-28 [302], HLA-20 [299], M30 [303], and VAR [304] were shown to protect TH+ cells in murine MPTP and 6-OHDA intoxicated models and increase DA content in the striatum. In addition to the 8-hydroxyquinoline chelator moiety, HLA-20, M30, and VAR also have the monoamine oxidase (MAO) inhibitor group propargyl, conforming bifunctional iron chelator/MAO inhibitor drugs. These molecules were demonstrated to chelate iron, decrease DA breakdown, and induce prosurvival factors through putative interactions with signaling components. Indeed, M30 was shown to upregulate protein levels of hypoxia inducible factor 1*α* (HIF-1*α*), through decreasing the activity of HIF-degrading enzyme HIF prolyl hydrolase [305–307]. As a consequence, many prosurvival genes controlled by HIF-1*α* were upregulated after M30 administration, including vascular endothelial growth factor, erythropoietin, enolase-1, transferrin receptor 1, heme oxygenase-1, inducible nitric oxide synthase, and glucose transporter 1 [307]. In addition, mRNAs for brain-derived neurotrophic factor, glial cell-derived neurotrophic factor, and three antioxidant enzymes (catalase, superoxide dismutase-1, and glutathione peroxidase) were also upregulated by M30 administration [307, 308]. Possibly, these later genes are activated through the propargyl moiety via induction of increased phosphorylation of protein kinase C, mitogen-activated protein kinase (MAPK/ERK), protein kinase B, and glycogen synthase kinase-3*β*s [304]. In addition, Naoi and Maruyama suggested that the propargyl moiety might stabilize the mitochondrial membrane through direct interaction with protein components of the mitochondrial permeability transition pore, leading to increasing levels of antiapoptotic Bcl-2 and Bcl-xL proteins [309]. Supporting the prosurvival effects of iron chelators, a recent study showed that M30 and other hydroxyquinoline-based iron chelators regenerate the neuritic tree in cultured DA neurons treated with sublethal concentrations of MPP+; in addition, M30 given orally regenerated nigrostriatal fibers mouse model after MPTP intoxication [310]. Following the multifunctional approach in iron chelation, others studies tested iron chelators with D2/D3 dopamine receptor agonists to attack the motor symptoms and the oxidative stress simultaneously in the MPTP and lactacystin PD models. Interestingly, the authors found that activation of D3 dopamine receptors was important for the protective effect of these molecules [311, 312].

Other studies reported that some phytochemicals evaluated in their capacity to confer neuroprotection in PD models acted through iron chelation [313]. Curcumin, a lyphenolic compound from* Curcuma longa* decreases the iron content in the SNpc of 6-OHDA lesioned rats and partially protects them from the decrease in the number of TH+ cells [314]. Moreover, ginkgetin, a biflavonoid from* Ginkgo biloba*, showed neuroprotection and attenuated the decrease in mitochondrial membrane potential in dopaminergic cell cultures [295]. In addition, ginkgetin enhanced the performance in the rotarod test and attenuated SNpc neuron lost in the MPTP mouse model [295].

Despite the promising character of the field, only the relatively old iron chelator deferiprone (DFP) has been tested in clinical trials for the treatment of PD. DFP is a small lipophilic molecule that is orally active since it crosses the intestinal and blood-brain barriers. DFP also permeates the cell and mitochondrial membranes, interchanging iron between mitochondria, cytoplasm, and extracellular apotransferrin, that is, not only chelating iron but also redistributing it [315]. The ability to “move” iron out of mitochondria is a very important property because, as discussed earlier, the mitochondrion has a prominent reactive iron pool and is the major ROS producer in the cell [28, 94, 316].

A pilot clinical trial of DFP in PD patients, tested with a design comparing the progression in iron content trough MRI and behavior alterations by the Unified Parkinson's Disease Rating Scale, was successful. Comparison between groups that began the treatment with a six-month difference (“early start” and “delay start” groups) showed significant improvement in the parameters in the “early start” group compared with the “delay start” group [317].

A possible drawback of putative iron chelating therapy is that chelators may facilitate the depletion of systemic iron, with severe consequences for other organs like the heart, the liver, and the hematopoietic system [286, 287]. The detected undesirable effects of iron chelation include neutropenia in a small percent of DFP-treated patients [317] and the possibility of high blood pressure resulting from the selective inhibition of peripheral MAO-A by the propargyl moiety of M30 and VAR [304]. Maneuvers designed to counteract these undesirable effects of iron chelation should be sought-after in futures studies.

Clioquinol, recently evaluated in clinical trials [318, 319], presented apparently neurotoxic properties at high doses. Indeed, clioquinol was indicated like the causative agent of subacute myelo-optic neuropathy (SMON) [320], DNA double-strands breaks induction [321], superoxide dismutase 1 inhibition [322], and nerve growth factor-induced Trk receptor autophosphorylation inhibition [323]. In addition, the clioquinol derivative PBT2 showed low effectiveness and in some cases adverse effects in a recently phase-2 trial for Huntington's disease [324].

Overall, the above evidence shows that iron chelation is a promising therapeutic approach to slow or rescue the neurodegenerative process of PD. The development of new chelators should consider characteristics to make them specific for cell type and effective at lower concentration than those actually in use. A high affinity for iron seems not to be relevant for neuroprotection [325] but as Mena et al. showed [172], mitochondrial targeting should enhance mitochondrial protection and neuroprotective capacity. In summary, the neuroprotective effects of iron chelation reported up to date are a stimuli for the development of new multifunctional iron chelators with blood-brain barrier permeability and mitochondrial targeting, with significant activity at pharmacological concentrations and devoid of noxious side effects.

## 10. Concluding Remarks

**Figure 3:**
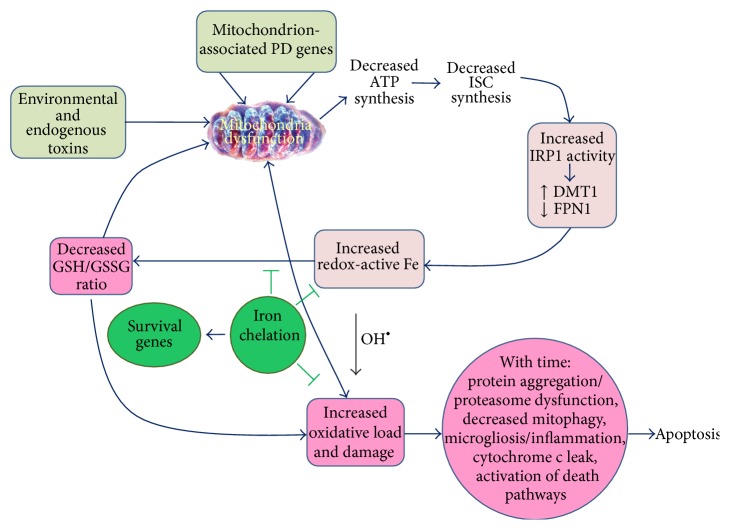
Mitochondrial dysfunction leads to iron accumulation and cell death. Mitochondrial dysfunction in PD, caused either by environmental or endogenous toxins or by genetic dysfunctions, results in decreased ATP and ISC synthesis. The lack of ISCs results in a false low iron signal and the spurious activation of IRP1. Activation of IRP1 results in increased redox-active iron levels mediated by increased expression of DMT1 and TfR1 and decreased expression of FPN1. Because of hydroxyl radical generation through the Fenton reaction, increased redox-active iron results in a decreased GSH/GSSG ratio and an increased oxidative load. The decrease in GSH further affects mitochondrial activity. With time, the increased oxidative load induces protein aggregation and saturation of the ubiquitin-proteasome system, further mitochondrial dysfunction, an inflammatory microenvironment, increased cytochrome c leak, and activation of death pathways. Iron chelation has been demonstrated to slow this cycle by decreasing iron-associated oxidative damage and by induction of cell survival and cell-rescue pathways. Environmental and endogenous toxins: paraquat, rotenone, MPTP, nitric oxide, 4-hydroxynonenal, advanced glycation end products, and aminochrome. Mitochondria-associated PD genes with mitochondrial dysfunction component: *α*-Syn, Parkin, PINK1, DJ-1, LRRK2, and ATP13A2.

The mitochondrion is the main intrinsic ROS producer in the cell and has an intensive traffic of iron due to the synthesis of ISCs and heme prosthetic groups. Because of the Fenton reaction, mitochondrial levels of ROS and iron need to be tightly regulated to avoid generation of the damaging hydroxyl radical. In both idiopathic and familial cases of PD, mitochondrial dysfunction, iron accumulation, and oxidative damage are commonly found in defective neurons. We propose that these three occurrences are causally linked (Figure 3). Mitochondrial dysfunction, product of endogenous or exogenous toxins, or genetic predisposition results not only in increased ROS production but also in decreased ISC synthesis and IRP1 activation. In turn, IRP1 activation results in iron accumulation and hydroxyl radical-mediated damage. These three events—mitochondrial dysfunction, iron accumulation, and oxidative damage—generate a positive feedback loop of increased iron accumulation and oxidative stress. Intervention at some of these three levels may retard the progression of the disease. Pharmacologically, this effect could be achieved with the use of multifunctional molecules with iron chelation capacity, since iron chelation has been linked to the protection against oxidative damage and the activation of prosurvival pathways.
